# Digging Deeper: In Search of the Mechanisms of Carbon and Nitrogen Exchange in Ectomycorrhizal Symbioses

**DOI:** 10.3389/fpls.2019.01658

**Published:** 2020-01-14

**Authors:** Emiko K. Stuart, Krista L. Plett

**Affiliations:** Hawkesbury Institute for the Environment, Western Sydney University, Richmond, NSW, Australia

**Keywords:** mutualism, symbiosis, nutrition, carbon sequestration, ecosystem modelling, carbon cycle, mycorrhizae

## Abstract

Symbiosis with ectomycorrhizal (ECM) fungi is an advantageous partnership for trees in nutrient-limited environments. Ectomycorrhizal fungi colonize the roots of their hosts and improve their access to nutrients, usually nitrogen (N) and, in exchange, trees deliver a significant portion of their photosynthetic carbon (C) to the fungi. This nutrient exchange affects key soil processes and nutrient cycling, as well as plant health, and is therefore central to forest ecosystem functioning. Due to their ecological importance, there is a need to more accurately understand ECM fungal mediated C and N movement within forest ecosystems such that we can better model and predict their role in soil processes both now and under future climate scenarios. There are a number of hurdles that we must overcome, however, before this is achievable such as understanding how the evolutionary history of ECM fungi and their inter- and intra- species variability affect their function. Further, there is currently no generally accepted universal mechanism that appears to govern the flux of nutrients between fungal and plant partners. Here, we consider the current state of knowledge on N acquisition and transport by ECM fungi and how C and N exchange may be related or affected by environmental conditions such as N availability. We emphasize the role that modern genomic analysis, molecular biology techniques and more comprehensive and standardized experimental designs may have in bringing cohesion to the numerous ecological studies in this area and assist us in better understanding this important symbiosis. These approaches will help to build unified models of nutrient exchange and develop diagnostic tools to study these fungi at various scales and environments.

## Introduction

**Figure 1:**
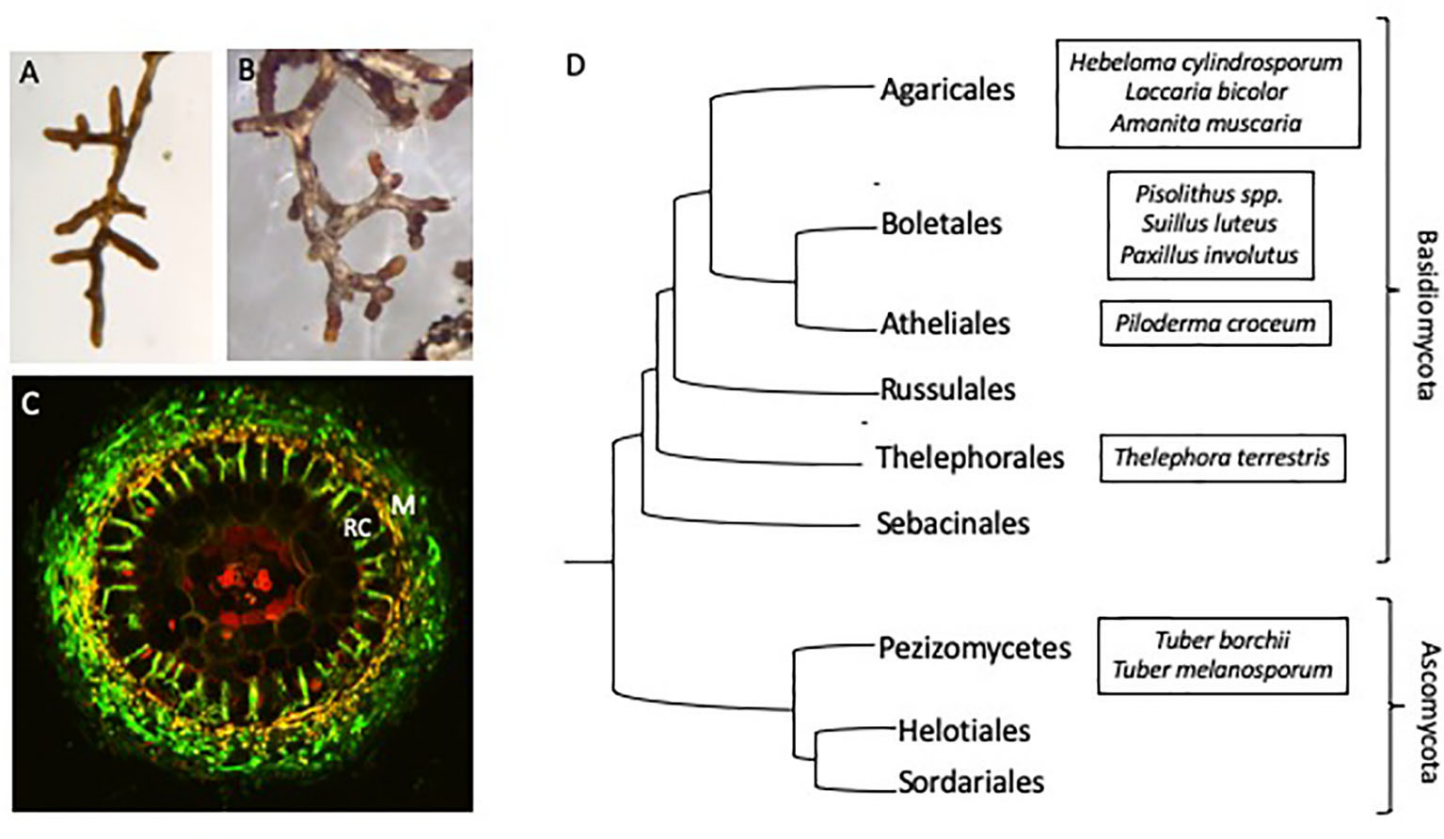
Physiology and phylogeny of ECM fungi **(A)** *Eucalyptus grandis* roots colonized by *Pisolithus microcarpus* and **(B)** *Pinus radiata* colonized roots showing the typical morphology of mycorrhizal root tips. **(C)** Cross section of a mycorrhizal root tip, showing the fungal mantle (M) surrounding the root and ingrowth of fungal hyphae between the root epidermal cells (RC). Fungal hyphae (green) is stained with WGA-FITC. **(D)** Phylogenetic diversity of the major ECM containing orders of fungi with selected model ECM species (adapted from Kohler et al., 2015 and Nagy et al., 2016).

Ectomycorrhizal (ECM) fungi are found throughout the world in association with the roots of most forest trees. While ECM fungi are thought to provide their hosts with a range of benefits, including drought (Jones and Smith, 2004; Rapparini and Peñuelas, 2014) and salinity tolerance (Hajiboland et al., 2010), they are primarily categorized as being useful for host nutrient acquisition (Read and Perez-Moreno, 2003; Smith and Read, 2008). These fungi colonize the lateral roots of host trees, forming interlacing mycelial structures that penetrate between and surround root epidermal cells (**Figures 1A**–**C**). This unique structure, called the Hartig net, provides a large amount of surface area between the two symbiotic partners and is the site of nutrient exchange. Carbon (C) resources from the host are transferred to the fungus in return for limiting nutrients, which the fungus can either access from beyond the nutrient depletion zone surrounding the host’s root system (Smith and Read, 2008) or release from immobilized sources normally inaccessible to the plant.

Ectomycorrhizal fungi are therefore essential contributors to forest ecosystem functioning. By forming a beneficial symbiotic relationship with the roots of 80% to 90% of all temperate and boreal forest trees (Read, 1991; Brundrett, 2004), these fungi drive forest soil processes, such as soil organic matter decomposition, nutrient cycling and C sequestration (Read and Perez-Moreno, 2003; Orwin et al., 2011; Zak et al., 2019). As recently reviewed by Becquer et al. (2019), ECM fungi have the ability to uptake and provide their hosts with a range of macronutrients, including phosphorus, potassium, calcium, magnesium, sulphur, and micronutrients, such as iron, zinc, copper, and manganese. However, they are recognized mostly for their provision of nitrogen (N) because it is the main growth-limiting factor in many forest ecosystems, particularly in the Northern hemisphere (Read et al., 2004; Toljander et al., 2006; Lin et al., 2017). Most of the N in forest soils is in organic form, bound up in soil organic matter or in leaf litter that accumulates on the forest floor (Read, 1991; Bruns et al., 2002). The mineralisation rates of these organic complexes are too slow for sufficient plant N nutrition, however, ECM fungi have some ability to decompose these organic complexes making ECM fungi important players in soil N cycling (Read, 1991; Lindahl et al., 2007; Bödeker et al., 2014; Lindahl and Tunlid, 2015; Shah et al., 2016).

Additionally, ECM fungi are thought to have a role in promoting soil C sequestration (Orwin et al., 2011; Cairney, 2012; Clemmensen et al., 2014). A substantial portion of plant photosynthate is sent belowground to ECM fungal partners (Smith and Read, 2008). While some of this photosynthate is returned to the atmosphere *via* fungal respiration or decay (Talbot et al., 2008), ECM fungi are believed to generally repress soil respiration (Gadgil and Gadgil, 1971; Gadgil and Gadgil, 1975; Averill et al., 2014; Averill and Hawkes, 2016) and thus increase C storage, though this effect may be highly dependent on the fungal species and on soil conditions (Fernandez and Kennedy, 2016; Baskaran et al., 2017).

Owing to their crucial role in soil biogeochemical processes and forest productivity, there is an increasing effort to incorporate mycorrhizal fungi into ecosystem modeling equations (Brzostek et al., 2014; Sulman et al., 2017). This effort is hampered, however, by a lack of knowledge concerning the actual contributions of ECM fungi to all of these soil processes and how soil conditions such as nutrient availability influence outcomes (Deckmyn et al., 2014; Koide et al., 2014). Indeed, while much research on C allocation to ECM fungi and the corresponding transfer of N to host tissues has been conducted, there is no generally accepted mechanism for the control of nutrient flux between the symbiotic partners. Additionally, research into the effects of soil nutrient availability on this exchange give variable and often conflicting results (see *Effect of N Concentrations in Soils*).

One of the potential contributing factors to the difficulties in generating a unified mechanism for C/N exchange is the complicated evolutionary history of ECM fungi. Ectomycorrhizal fungi are a diverse group of asco- and basidiomycetes, with over 20,000 species that evolved independently from 60 to 80 saprotrophic ancestral lineages (**Figure 1D**; Hibbett et al., 2000; Rinaldi et al., 2008; Tedersoo and Smith, 2013; Martin et al., 2016). Comparatively, within arbuscular mycorrhizal (AM) fungi, approximately 240 species are described, likely descending from a single ancestral lineage (Brundrett, 2002; Lee et al., 2013; Selosse et al., 2015). While all ECM fungi retain in common a unique symbiotic structure and the ability to exchange nutrients with their host, traits such as host and climate range, hyphal growth characteristics and the ability to source soil nutrients vary widely between species (Clemmensen et al., 2014; Pellitier and Zak, 2018). Thus, one simple model of C allocation and N transfer may not suffice for ECM fungi as a whole.

Here in this review we summarize the current state of research concerning the acquisition and transport of soil N by ECM fungi and the C for N trade with their hosts, and consider some of the challenges in the field and opportunities for future research. In particular, we emphasize the role that genome sequencing projects and functional characterization using molecular biology techniques may have in reconciling the collected field data and building mechanisms to model nutrient exchange dynamics.

## Nutrient Uptake and Transport

In order to supply plant tissues with N, the ECM fungus must first acquire the nutrient from its environment. Ectomycorrhizal fungi encode a number of transporters for the acquisition of nitrate and ammonium ions from soil, as well as a suite of enzymes and transporters necessary for utilizing organic N sources (Casieri et al., 2013; Nehls and Plassard, 2018; Becquer et al., 2019). Many of these transporters have been well characterized in model ECM species; however, a number of important questions remain unanswered in this area of research. For example, we still need to identify transporters that negotiate the exchange of nutrients at the plant-fungal interface and determine the extent to which fungal transporters are conserved amongst different species of ECM fungi, or how they are regulated under different soil and nutrient conditions. The ability to use N from different organic sources is, for example, particularly variable between species (Courty et al., 2005; Buee et al., 2007; Pena et al., 2013) and even within species (Anderson et al., 1999; Sawyer et al., 2003; Guidot et al., 2005). This knowledge gap presents an excellent opportunity for future studies to refine this area of science.

### Inorganic N Import

Ammonium is generally the preferred inorganic N source of ECM fungi, as nitrate is immediately reduced to ammonium after uptake and thus requires more energy to assimilate (Plassard et al., 2000). Ammonium importers AMT1, AMT2 and AMT3 have been functionally characterized in several ECM fungal species, including *Hebeloma cylindrosporum* (Javelle et al., 2001; Javelle et al., 2003), *Tuber borchii* (Montanini et al., 2002) and *Amanita muscaria* (Willmann et al., 2007). Homologues of these genes have also been identified in many other ECM fungal genomes from transcriptomic studies (Lucic et al., 2008; Hortal et al., 2017). AMT1 and AMT2 have been characterized as high affinity ammonium transporters in different fungi, and their expression is most up-regulated in low ammonium conditions (Javelle et al., 2003; Willmann et al., 2007). Nitrate transporters, such as LbNRT2 in *Laccaria bicolor* (Kemppainen et al., 2010) and HcNRT2 in *H. cylindrosporum* (Jargeat et al., 2003), are also present in ECM genomes, allowing for growth on nitrate. Transcriptional regulation of nitrate transporters is usually mirrored by the corresponding regulation of nitrate reductase enzymes, required to assimilate the transported nitrate and reduce it to ammonium (Kemppainen et al., 2009; Kemppainen et al., 2010). The nitrate uptake pathway has been found to be down-regulated by the presence of ammonium, but not by the presence of organic N sources, allowing for the simultaneous uptake of nitrate and organic N (Jargeat et al., 2003; Kemppainen et al., 2010).

### Organic N Acquisition and Import

Ectomycorrhizal fungi all evolved from saprotrophic ancestors, and hence retain the ability to decompose organic matter (Kohler et al., 2015; Shah et al., 2016). However, analysis of their genomes displays a strikingly reduced complement of enzymes required for the decomposition of complex organic matter, particularly cell-wall decomposing enzymes (Kohler et al., 2015; Lindahl and Tunlid, 2015). As a result, while most ECM fungi are able to survive on and use organic material, they do so with a reduced capacity as compared to saprotrophs (Shah et al., 2016).

Despite their commonly held ability to decompose organic matter, ECM fungi have different mechanisms and efficiencies of decomposition stemming from their diverse ancestral origins. In fact, there is a high degree of variability in the type and number of genes retained from saprotrophic ancestors amongst investigated ECM fungal species (Kohler et al., 2015) and thus a high variation in decay ability (Pellitier and Zak, 2018). Most ECM fungi appear to use primarily oxidative mechanisms, such as Fenton chemistry, to break down soil organic matter (Rineau et al., 2012; Bödeker et al., 2014; Martin et al., 2016; Shah et al., 2016; Op De Beeck et al., 2018). Fenton chemistry originates from brown-rot saprotrophic ancestors, which use these mechanisms to biodegrade lignified plant cell wall material and obtain nutrition (Eastwood et al., 2011). These mechanisms are considered to be more energetically efficient than the production of secreted class II peroxidases characteristic of white-rot fungi (Eastwood et al., 2011). However, unlike brown-rot saprotrophs, ECM fungi do not appear to use significant amounts of the broken-down organic matter as a C source. Instead, relying on host supplied C, they assimilate primarily N from these organic molecules (Treseder et al., 2006; Lindahl et al., 2007; Le Tacon et al., 2015; Martin et al., 2016; Shah et al., 2016). Meanwhile, several other ECM species from the order Agaricales evolved from white-rot fungi and a few, including *Cortinarius* species, retained the Class II peroxidases needed for complete organic matter decomposition (Bödeker et al., 2009; Kohler et al., 2015; Lindahl and Tunlid, 2015; Martin et al., 2016).

Ectomycorrhizal fungi also secrete an assortment of peptidases to utilize proteins within the soil (Nehls et al., 2001; Müller et al., 2007; Shah et al., 2013; Rineau et al., 2016) and encode a number of amino acid, oligopeptide, and dipeptide transporters to take up the resulting small peptide products (Nehls et al., 1999; Benjdia et al., 2006; Lucic et al., 2008; Casieri et al., 2013). Expression of these organic N transporters and secretion of peptidase are usually reduced in the presence of ammonium, indicating the fungal preference for uptake of the more easily accessible ammonium from soil (Avolio et al., 2012; Bödeker et al., 2014). Further, the decay of organic matter *via* peptidase secretion to acquire N may be triggered by glucose availability (Rineau et al., 2013); thus, fungi in a mycorrhizal association and receiving sugars from a host plant would be more likely to source organic N from the soil. As in the decay of more complex organic matter, the ability to produce these enzymes and utilize the organic matter varies considerably by ECM species (Courty et al., 2005) and soil conditions (Buee et al., 2007).

### Transfer of N to Plant Tissues

Once taken up into the fungal mycelium, N is metabolized, often stored, and eventually transported to other fungal cells (see Nehls and Plassard, 2018 for a review on this subject). Some of this N makes its way to ECM root tips to be passed on to the plant. In order to pass from the fungal cell to the plant cell, N has to transverse two membranes. This necessitates coordination between the expression and activity of fungal exporters and that of the corresponding plant importers. The up-regulation of plant ammonium importer expression in mycorrhizal root tips has led to the theory that ammonium is the principle form of N transferred between the two organisms (Selle et al., 2005; Chalot et al., 2006; Couturier et al., 2007; Chalot and Plassard, 2011; Nehls and Plassard, 2018). Evidence of amino acid transport and the exchange of organic compounds in the apoplastic space also exists, however, with fungal amino acid exporters being up-regulated in mycorrhizal root tips colonized by *L. bicolor* or *Pisolithus microcarpus* (Larsen et al., 2011; Hortal et al., 2017). While most of the research focus is on the regulation of ‘classical’ N transporters in ECM tissues, aquaporins (Dietz et al., 2011) and voltage-dependent cation channels (Chalot et al., 2006) are also up-regulated in expression in mycorrhizal tissue and have been shown to transport ammonium. Overall, while the transport of N from fungal to plant tissues is certainly an accepted process, neither the form of N transported nor the specific fungal or plant transport mechanisms used are fully known.

### Carbon Transfer

While estimates vary, a third or more of tree photosynthate may be directed to ECM associates (Nehls et al., 2010). It is not surprising, therefore, that colonization results in an up-regulation of tree C metabolic and photosynthetic pathways (Nehls et al., 2007; Larsen et al., 2011). These sugars, primarily sucrose, are transported to colonized root tissues and into the apoplastic space between plant and fungal cells using a variety of plant transporters (Hennion et al., 2019). Plants encode a number of sucrose transporters (SUTs), involved in the long-distance transport of sugars in the plant (Doidy et al., 2012). The main sucrose cleavage products, glucose, and fructose, as well as other mono-saccharides, are transported between cellular compartments with mono-saccharide transporters (MSTs) or SWEET transporters (Sugar Will Eventually be Exported Transported; Chen et al., 2010). While most SWEET transporters move monosaccharides, some SWEET transporters, particularly AtSWEET11 and 12, are able to transport sucrose from mesophyll cells (Chen et al., 2012; Doidy et al., 2012). It is not currently known which of the many plant sugar transporters is responsible for the export of sugar to the fungal symbiont.

It is generally understood that plants secrete sucrose into the plant-fungal interface. However, while several monosaccharide importers with increased expression in mycorrhizal root tips have been identified in ECM fungi (Nehls et al., 2007; López et al., 2008), sucrose transporters are notably absent in most ECM fungal genomes (Salzer and Hager, 1991; Martin et al., 2008). These fungi also commonly lack the secreted invertases required to cleave sucrose into usable mono-saccharides (Martin et al., 2008), making sucrose a non-viable source of nutrition. The fungus is thus reliant on the plant, not only for sugars, but additionally for invertases to break down sucrose into a useable form (Salzer and Hager, 1991). Gene expression analyses do indeed reveal the increased expression of plant invertases (Wright et al., 2000; Nehls et al., 2010) at the plant-fungal interface, but interestingly, also the increased expression of a number of plant monosaccharide importers. This may provide the plant with a mechanism to control the loss of sugar resources to the fungus and represent a method for the plant to select for desirable partners by increasing their access to sugar, or conversely, discourage less helpful partners (Nehls et al., 2010). However, this hypothesis remains theoretical as the mechanisms controlling C flux from host to fungus are as yet largely unknown.

### Section Summary

While knowledge of the nutrient uptake, transport, and metabolic pathways in ECM fungi is progressing well (Nehls and Plassard, 2018), there are still many missing pieces in the current research models, particularly concerning the regulation of transport at the mycorrhizal interface. This highlights the need for research efforts in the identification and functional characterization of these transporters and of the forms of nutrients exchanged between plant and fungus. Additionally, research into how evolutionary history, genotype, and abiotic factors affect the regulation and activity of these transporters is needed. Improved knowledge about these transporters would allow for better predictions of where nutrients are going belowground, particularly if their regulation is found to correlate to nutrient flux. Such knowledge could lead to the development of a simple diagnostic tool to approximate the rates of C/N exchange between partners, which is the subject of the next section.

## Factors Controlling Carbon and N Flux

### Defining C and N Flux

Having considered the transporters negotiating the exchange of nutrients between ECM fungus and host, we now turn our attention to questions of quantity. What factors determine the amount of nutrition received by each partner in this relationship? Despite its importance, knowledge on what moderates the amount of C and N exchanged, or C/N flux, in ECM symbioses is lacking, particularly in comparison to that of C and P exchange in AM symbioses (Garcia et al., 2015). Many studies on the effects of ECM colonization look at plant growth outcomes, rather than direct nutrient exchange. These studies reveal a variety of growth responses in the plant, ranging from positive to negative (Johnson et al., 1997; Jones and Smith, 2004; Corrêa et al., 2012). While it is often assumed that negative growth responses in plants to ECM fungi occur as a result of excessive C drain compared to the nutritional benefit received, particularly at high N concentrations, C loss has been determined in some studies to be of little or no cost to the plant, so these negative growth results could as easily be due to immobilization of N resources by the ECM fungus (Corrêa et al., 2012). In order to elucidate the causes of these growth outcomes and better understand nutrient cycling and movement in forest environments, a focus on the relative flow rates, or “fluxes,” of N and C, and the mechanisms behind their control, is needed. While the measurement of both N and C flux can be challenging, especially in field studies (Hobbie and Hobbie, 2006; Le Tacon et al., 2015), it is important in understanding the mechanisms behind the bidirectional transfer of nutrients that upholds this association.

Several methods, ranging in their accuracy as a proxy for nutrient flux, have been deployed in this area of research. Earlier approaches to understanding this exchange involved measuring absolute C and N amounts in the tissues of plants and fungi and used changing C/N ratios as a proxy for their access to nutrients (Colpaert et al., 1996; Nilsson and Wallander, 2003). Currently, a common method of measuring ECM nutrient flux is the use of isotopic labeling. The addition of either ^13^C to trees (usually as carbon dioxide; e.g. Högberg et al., 2010) or ^15^N compounds to soil (e.g. Hasselquist et al., 2016) allows for tracking of the uptake and movement of these nutrients over time. The application of stable ^15^N is not without disadvantages. It is most suited to shorter-term experiments, and the addition of the usually inorganic label may alter soil chemistry or disturb existing microbial relationships. Additionally, it is very difficult to eliminate the possibility of direct uptake of the label by the tree outside of the mycorrhizal pathway. The use of natural abundances of ^15^N, possible due to a bias in ECM fungi to preferentially give ^14^N to the plant (Hobbie and Colpaert, 2003), eliminates some of these issues, but sacrifices sensitivity and thus is best suited to long term studies (Hobbie and Hobbie, 2006; Högberg et al., 2011).

While these techniques are all capable, with varying levels of accuracy and ecological relevance, of quantifying the movement of C and N between plant and fungal partners, the challenge remains to link this data to a mechanism. Future studies could incorporate these physiological techniques to quantify nutrient movement with transcriptomic analyses and other molecular techniques to begin to answer questions concerning the role of each partner in this nutrient trade and causative factors behind nutrient flux (Johnson et al., 2012). Further, an increased effort to harmonize the different methods of measuring C for N exchange would allow for more meaningful comparisons between data sets.

### Coupling of C and N Flux

A popular theory behind the regulation of C and P exchange in AM symbioses is the ‘reciprocal rewards’ concept, whereby plant photosynthate is preferentially transferred to fungal symbionts that provide more nutrients (Kiers et al., 2011; Fellbaum et al., 2012; Fellbaum et al., 2014). This theory has by extension led to the consideration that C allocation to ECM fungi may follow a similar pattern with the amount of C delivered being related to the amount of N sourced by the fungus (Casieri et al., 2013). This theory is attractive in that it provides a mechanism for the stabilization of the mutualism; provision of N to a host is a substantial cost to the ECM fungus, thus if it were not compensated for in some way, individuals providing less host benefit would be expected to flourish at the expense of their more helpful counterparts (Hoeksema and Kummel, 2003; Bever et al., 2009; Moeller and Peay, 2016).

A recent study by Bogar et al. (2019) lent support to this theory. In a *Pinus muriata-Suillus brevipes* symbiosis studied in an artificial soil medium, mycorrhizal root tips that contained higher N concentrations received more plant C. However, this was also true of non-mycorrhizal root tips, suggesting that the plant may simply detect sources of high N and allocate C toward those sources. Similarly, Kaiser et al. (2017) found that for *Fagus sylvatica*-associated fungi, ^15^N enriched ‘hotspots’ within the fungal mycelium were also always highly enriched in ^13^C from the plant, suggesting that reciprocation was occurring. Another study using birch colonized by *Paxillus involutus* demonstrated a reciprocal exchange of C and N under various nutrient conditions (Kytöviita, 2005).

Meanwhile, many other studies have concluded that N and C flux is neither reciprocal nor related (Corrêa et al., 2008; Albarracín et al., 2013; Valtanen et al., 2014; Hasselquist et al., 2016). 2012; Corrêa et al. (2008) found that *Pinus pinaster* trees allocate C to *Pisolithus tinctorius* fungal symbionts whenever it is produced in excess, and continue to allocate that C even when the amount of N provided by the fungus is reduced (Corrêa et al., 2008). Näsholm et al. (2013) found that in N limited boreal forest soils, trees increased C allocation to ECM fungi, but this was not reciprocated with an increase in N transfer. In an *in vitro* system studying the *Eucalyptus grandis-Pisolithus microcarpus* association, Hortal et al. (2017) did not find a correlation between N and C allocation; in fact, *E. grandis* allocated the most C to the *Pisolithus* isolate that provided the least N. However, when a less beneficial *P. microcarpus* isolate was in competition with a more beneficial isolate, its ability to colonize the root system was reduced, coinciding with an up-regulation of plant defence pathways in only those roots associated with the less beneficial isolate. This indicates that the plant may not necessarily reward symbionts providing better nutritional benefit with proportionally more C, but may sanction less beneficial isolates through restricted root access (Simard et al., 2003; Kemppainen et al., 2009; Hortal et al., 2017).

Together, these studies suggest that while C delivery may under some circumstances be driven by the N content of ECM roots tips, the exchange of nutrients does not always follow a reciprocal exchange. There are, necessarily, other factors determining the transfer of nutrients yet to be discovered. It has been suggested that rather than the N status of a particular root tip, the N status of the tree leaves may be a more important driving factor in C allocation to roots (Nilsson and Wallander, 2003; Corrêa et al., 2008). The particular host and fungal genetic combination and soil abiotic conditions likely plays a role in C/N exchange dynamics as well. Research into the particular mechanisms and signaling events driving C and N trade is needed to better understand how this occurs and to be able to make better predictions of mycorrhizal outcomes in different environments.

### Effect of N Concentrations in Soils

The benefit of ECM fungi to tree growth is usually seen most apparently in severely N limited soils (Read et al., 2004; Smith and Read, 2008). For example, a synthesis of global research considering the effect of elevated carbon dioxide concentration on tree growth has shown that growth is limited by N availability, but the addition of ECM fungi can overcome this barrier (Terrer et al., 2016). In opposition to this view, however, is the idea that ECM fungi may not alleviate N limitation in forests, but rather form part of the problem. As ECM fungi themselves require N for their own growth, in N limited conditions they can “hoard” the available N and actually increase the N limitation of the forest soils (Näsholm et al., 2013; Franklin et al., 2014). Compounding the problem, under N limited conditions trees send more C belowground, resulting in more fungal biomass and an increased fungal demand for N (Högberg et al., 2003; Nilsson and Wallander, 2003; Treseder, 2004; Högberg et al., 2010). The balance between ECM fungi as positive growth regulators under limited N or as drivers of that N limitation likely is the result of a complex interaction between tree and fungal genotypes, soil nutrient conditions, and the nutritional needs of each organism (Alberton and Kuyper, 2009).

Studies on the effect of N addition to forest soils on ECM N transfer also show some variable responses. With increasing soil N concentration, some studies demonstrate that ECM fungi begin to mobilize stored N and transfer it to the host tree (Högberg et al., 2011; Näsholm et al., 2013; Hasselquist and Högberg, 2014) while other studies saw an opposite trend (Albarracín et al., 2013). Overall, however, the research overwhelmingly indicates that increased N has a negative impact on ECM communities. N fertilization results in reduced species richness, plant colonization levels, sporocarp production, and mycelial growth in soils, as well as a community shift to more nitrophilic ECM species and saprotrophs (Lilleskov et al., 2002; Nilsson and Wallander, 2003; Parrent et al., 2006; Allen et al., 2010; Pardo et al., 2011; Morrison et al., 2016; Corrales et al., 2017; Averill et al., 2018). Thus, while ECM fungi may, in some circumstances, drive N limitation, that N limitation may be important to their continued persistence and dominance in forest ecosystems. We are, however, left again with no unified theory as to how soil N availability may affect the nutritional benefit received by the host tree from its associated ECM fungi.

### Reconciling the Data

Each of the studies on C and N flux referenced above are highly valuable, however, research has still not developed a clear picture of how C and N exchange are related, either to each other, or to the soil N availability. While on the surface, the results of these studies may appear to be in conflict with one another, it is perhaps more useful to consider them as different perspectives on the same question as they consider N for C exchange in a variety of different environmental conditions and using different ECM fungi and host systems. It is possible, given the high degree of genetic variability in each fungal-host combination, that one central mechanism of C for N exchange is not attainable. From this view, more studies are required to complete the picture and determine where the consistencies lie and where we must simply expect variability. Additionally, ECM fungi contribute in many ways to plant health and nutrition beyond N and any one of these factors may also impact nutrient exchange. This highlights the need to complement observational and nutrient tracing studies with an understanding of the molecular mechanisms driving nutrient transfer.

## Future Research Directions

**Figure 2:**
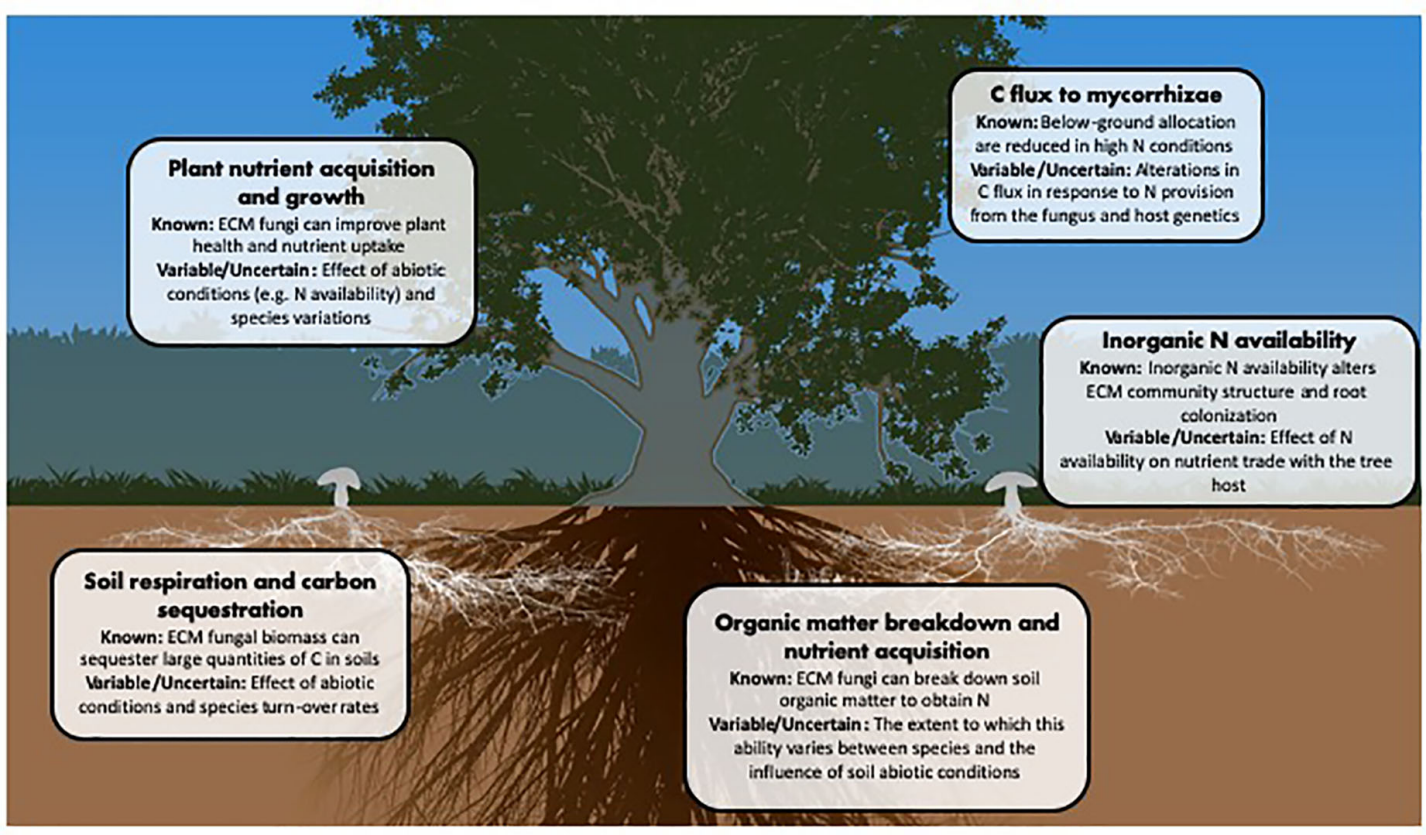
Major ecosystem parameters affected by or affecting ECM fungi.

ECM fungi affect numerous elements of forest ecosystem functioning and, while progress has been made towards understanding their general contributions to these processes, there remain many unknowns (**Figure 2**). Ecosystem models for nutrient movement incorporating mycorrhizal fungi have seen an increase in predictive power (Brzostek et al., 2014; Sulman et al., 2017; Keller and Phillips, 2019), however, these models are usually limited by assigning a mycorrhizal type as either ‘arbuscular’ or ‘ectomycorrhizal’. It has been suggested that these models could be improved by further dividing ECM fungi into subgroups based on their ecological function (Zak et al., 2019) or taxon-specific traits (Pena and Polle, 2014; Fry et al., 2019). Fungal growth type and foraging strategy may, for example, be simple parameters for ECM fungal classification, as both relate to the C demand of the fungus and its ability to find and source soil nutrients (Agerer, 2001; Clemmensen et al., 2014). Others have suggested that fungal C cost per unit N provided may be useful in characterizing the association (Corrêa et al., 2008; Agren et al., 2019); however, this is a complicated measure that may change dramatically based on external situations, or even over the lifetime of the mycorrhizal association (Nehls et al., 2016).

The limitation of such an approach is that they are currently relying on a relatively narrow set of model systems—systems that may not best portray the diversity of ECM fungal responses within natural ecosystems (Johnson et al., 2012; Zak et al., 2019). As discussed above, due to the fact that ECM fungal species do not share a single common ancestor, they may in reality have as many differences in function as those traits that they share. Supporting this claim, in a recent comparison of ECM genomes it was found that 7% to 38% of genes induced by symbiosis were species-specific (Kohler et al., 2015) while other studies have found that different species of ECM fungi colonizing the same host under the same conditions elicit different outcomes in terms of both nutrient exchange (Rincón et al., 2007; Alberton and Kuyper, 2009; Jones et al., 2009; Pena and Polle, 2014) and transcriptomic response (Peter et al., 2016). These differential responses can even be found between different fungal isolates of the same species (Plett et al., 2015a; Hazard et al., 2017; Hortal et al., 2017). Likewise, a single ECM fungus can also exhibit unique responses to different hosts (Plett et al., 2015b). Finally, the outcomes of ECM symbioses are affected by many diverse abiotic factors such as N availability, the amount and complexity of organic material in the soil (Plassard et al., 2000), or season (Högberg et al., 2010). Thus, we need to add to the range of studied ECM fungal lineages and abiotic conditions before we can gain a better ability to understand the diverse mechanisms by which ECM fungal communities drive nutrient cycling in forests.

Genomic sequencing and analysis will likely prove extremely important in this last aim, allowing for the assessment of common genetic features between groups of fungi. Linking of common genetic signatures to symbiosis-related outcomes could allow for a more rapid assessment of the likely contribution of an ECM fungus to plant health and nutrient cycling. However, this abundance of genomic information must be accompanied by more detailed molecular studies to elucidate the functions of various genes as many do not have a known function (Martin and Nehls, 2009; Plett et al., 2011). With special relevance to the topics discussed here, there are still large gaps in our knowledge concerning the specific transporters used by both fungi and hosts to transfer nutrients at the plant-fungal interface, particularly in the area of sugar transport. Increased understanding of the transporters involved, and related metabolic proteins, and how their regulation affects nutrient trade between partners would provide more accessible tools to assess efficiency of nutrient trading.

The literature would also suggest that the role of the plant host in determining mycorrhizal outcomes cannot be overlooked. Some manners by which host plants may be able to regulate associations with ECM fungi are by controlling C resources allocated to the ECM symbiont, restricting root colonization by an unhelpful symbiont, or even terminating or sloughing off mycorrhizal root tips with undesirable symbionts (Nehls et al., 2016). These mechanisms need further investigation. Like ECM fungi, host trees have a broad range of genetic diversity. Considering a wide variety of host trees, therefore, is necessary to determine the specificity of these mechanisms and the role of host genotype in symbiotic outcomes.

Finally, as we unravel the mechanisms underlying ECM symbioses at the molecular scale, we will need to incorporate the effect of biotic complexity at the ecological scale. While the ECM symbiosis is often thought of as an intimate relationship between a plant and a fungus, the natural habitats of ECM associations are, in fact, hugely complex, involving a wide array of interactions between each of the symbiotic partners and neighboring plants and fungi. Thus, findings at the one-on-one interactions level may not be replicated in the larger context (Kennedy, 2010). Therefore, substantial amounts of data will be needed in order to understand how all of these biotic factors affect a given plant-fungal combination in the field. This can be achieved, in part, from increased use of fully factorial experimental designs that incorporate multiple species.

In conclusion, as a whole, ECM fungi have been proposed as key players in nutrient cycling and as essential in equipping trees to survive and adapt to changing abiotic conditions (Lau et al., 2017). However, our ability to exploit or even predict the benefit of these fungi hinges on a greater understanding of their diversity, function, and contribution to nutrient cycles and forest health. Advances in molecular biology and genomic tools grant us the potential to dig deeper and to exploit the wealth of information from ecosystem scale studies and pot experiments and translate this into mechanistic understanding and new tools to better understand this important symbiotic system.

## Author Contributions

ES and KP envisioned and wrote the manuscript.

## Conflict of Interest

The authors declare that the research was conducted in the absence of any commercial or financial relationships that could be construed as a potential conflict of interest.
